# The rapid change in mental health among college students after introduction of on-campus quarantine during the 2022 Shanghai COVID-19 lockdown

**DOI:** 10.3389/fpubh.2023.1132575

**Published:** 2023-05-05

**Authors:** Dongni Ma, Yifang Kuang, Zhaohui Lan, Suhua Zeng, Yi Li, Mengnan Shang, Ru-Yuan Zhang, Binglei Zhao, Weidong Li

**Affiliations:** ^1^Bio-X Institutes, Key Laboratory for the Genetics of Development and Neuropsychiatric Disorders (Ministry of Education), Shanghai Key Laboratory of Psychotic Disorders, and Brain Science and Technology Research Center, Shanghai Jiao Tong University, Shanghai, China; ^2^Institute of Psychology and Behavioral Science, Antai College of Economics and Management, Shanghai Jiao Tong University, Shanghai, China; ^3^WLA Laboratories, World Laureates Association, Shanghai, China; ^4^SJTU Paris Elite Institute of Technology, Shanghai Jiao Tong University, Shanghai, China; ^5^Shanghai Mental Health Center, School of Medicine, Shanghai Jiao Tong University, Shanghai, China; ^6^Global Institute of Future Technology, Shanghai Jiao Tong University, Shanghai, China

**Keywords:** COVID-19, mental health, depression, college students, quarantine

## Abstract

**Objectives:**

Among the various impacts of disasters in terms of emotions, quarantine has been proven to result in significant increases in mental health problems. Studies of psychological resilience during outbreaks of epidemics tend to focus on long-term social quarantine. In contrast, insufficient studies have been conducted examining how rapidly negative mental health outcomes occur and how these outcomes change over time. We evaluated the time course of psychological resilience (over three different phases of quarantine) among students at Shanghai Jiao Tong University to investigate the influence of unexpected changes on college students.

**Methods:**

An online survey was conducted from 5 to 7 April 2022. A structured online questionnaire was administered using a retrospective cohort trial design. Before 9 March (Period 1), individuals engaged in their usual activities without restrictions. From 9 to 23 March (Period 2), the majority of students were asked to remain in their dormitories on campus. From 24 March to early April (Period 3), restrictions were relaxed, and students were gradually allowed to participate in essential activities on campus. We quantified dynamic changes in the severity of students’ depressive symptoms over the course of these three periods. The survey consisted of five sets of self-reported questions: demographic information, lifestyle/activity restrictions, a brief mental health history, COVID-19-related background, and the Beck Depression Inventory, second edition.

**Results:**

A total of 274 college students aged 18–42 years (mean = 22.34; SE = 0.24) participated in the study (58.39% undergraduate students, 41.61% graduate students; 40.51% male, 59.49% female). The proportion of students with depressive symptoms was 9.1% in Period 1, 36.1% in Period 2, and 34.67% in Period 3. Depressive symptoms increased notably with the introduction of the quarantine in Periods 2 and 3. Lower satisfaction with the food supplied and a longer duration of physical exercise per day were found to be positively associated with changes in depression severity in Periods 2 and 3. Quarantine-related psychological distress was more evident in students who were in a romantic relationship than in students who were single.

**Conclusion:**

Depressive symptoms in university students rapidly increased after 2 weeks of quarantine and no perceptible reversal was observed over time. Concerning students in a relationship, ways to take physical exercise and to relax should be provided and the food supplied should be improved when young people are quarantined.

## 1. Introduction

Outbreaks of severe epidemics pose a major challenge to psychological resilience. The coronavirus disease 2019 (COVID-19) was declared a public health emergency (1) in 2020 and threatened both physical and mental health. The negative emotions of people in the worst-hit areas were compounded by fear of infection, inadequate supplies, suspension of social public services, and unexpected social quarantine (2, 3). Among the various impacts of disasters in terms of emotions, sudden social quarantine has been proven to result in a significant increase in mental health problems.

Studies of people who were isolated from social contact during the COVID-19 outbreak showed significant psychological consequences in terms of posttraumatic stress (4), anxiety (5–7), and depression (5, 8, 9). Compared to the general population, university students are more prone to mental health problems (10). Multifaceted issues, including study-related stress, social pressures, financial difficulties, and feelings of isolation threaten their mental health. Investigations of college or university students in China, France, and the United States have shown that the COVID-19 quarantine has had a non-trivial effect on university students’ mental health (11–13).

The on-campus quarantine examined in this study had two key characteristics. First, there were attempts to emulate normal campus life. In other words, students continued to follow their normal curricula (online) and to engage in research activities with well-organized, uniform logistical support from the university. Second, there were features of a highly strict quarantine. Students were required to comply with various regulations and to remain in cramped dormitory conditions for 2 weeks or more. Understanding the implications and associated risk factors of such a complex regulation process is critical for the development of an appropriate emergency policy and for the promotion of wellbeing among students in on-campus quarantine. To date, researchers from France (13) and the United States (14, 15) have mainly focused on participants who were asked to stay at home during the pandemic. Quarantine in these studies refers to a less strict physical quarantine or merely to maintaining social distance, without compulsory separation from family or loved ones. While there have been prior studies of mental health issues among Chinese university students under stringent quarantine (16–18), these have not assumed a unified on-campus environment. Instead, these studies have examined citywide or countrywide data, in which the targeted quarantine situation varies greatly from individual to individual.

Here, we studied the time course of psychological resilience over three phases of quarantine at Shanghai Jiao Tong University (SJTU) upon the release of strict on-campus quarantine in order to investigate the influence of this unexpected change on university students. Students were required to stay in their dormitories and not to leave their rooms for a period of weeks. The aim of this study was to examine the mental health of college students during this campus quarantine in China and to offer some foundational evidence for the provision of psychological interventions for college students.

## 2. Methods

### 2.1. Participants

All undergraduate or graduate students at SJTU were regarded as potential participants and were asked to voluntarily participate in the survey through the network platform. A total of 276 students (111 male, 163 female) participated in the survey; of these responses, two questionnaires were excluded from follow-up analysis due to incomplete data. Therefore, the final survey data consisted of questionnaire responses from a total of 274 participants (response rate = 99.28%) aged between 18 and 42, of whom 160 were undergraduate students and 114 were graduate students.

### 2.2. Procedure

A survey link was placed on^1^ and sent to various university departments, including computer science, agriculture, and medicine; the data were collected from 5 to 7 April 2022. All participants were informed of the details of the study and provided informed consent. The study was approved by the ethics review committee for human-related scientific and technological research at SJTU.

### 2.3. Quarantine details and area

A structured online questionnaire was administered using a retrospective cohort trial design. Before 9 March (Period 1), individuals engaged in their usual activities without restrictions. The campus was locked down on 9 March. From the 9 to 23 March (Period 2), the majority of students were asked to stay in their dormitories on campus, and three meals were delivered daily by faculty members. The average area available for movement within the dormitory suite was approximately 30 square meters. From 24 March to early April (Period 3), restrictions were relaxed, and students were gradually allowed to engage in essential activities, but were still not allowed to leave the campus. The campus covers an area of 3,092,500 m^2^. We quantified dynamic changes in depression severity during the campus lockdown.

### 2.4. Measurement instruments

A structured online questionnaire was administered, in which depression symptoms and their associated potential risk factors (e.g., daily physical exercise time) were assessed during three periods of time. The survey contained a total of 41 questions (20 demographic and health-related questions, and 21 BDI-II questions). Demographic and health-related questions included demographic information, a brief mental health history, lifestyle/activity restrictions under campus lockdown, and COVID-19-related background.

#### 2.4.1. Demographic information

Demographic information included gender (male or female), age, level of study (undergraduate or graduate), and relationship status (with or without a partner).

#### 2.4.2. Brief mental health history

Three questions were asked about the respondent’s and their family’s history of depression, including whether their family had mental health issues, whether they had been diagnosed with depression, and their medication status (whether they were taking medication and the medication they were using).

#### 2.4.3. Lifestyle/activity restrictions under campus lockdown

This series of questions explored changes in the participant’s lifestyle and the levels of activity restriction across three different periods. These included self-reported ways of easing stress (connecting with loved ones, mentors, or psychiatrists for help; searching the internet; playing games; exercising; listening to music; etc.); the frequency with which they exercised, played video games, and communicated with loved ones or parents (scores ranged from 0 to 5, with 0 reflecting the lowest frequency and 5 the highest); their satisfaction with the food supplied (scores ranged from 0 to 5, with 0 reflecting the lowest level of satisfaction and 5 the highest); and the level of activity restriction they experienced.

#### 2.4.4. COVID-19-related background

Two questions were asked in relation to the COVID-19 pandemic. These included whether or not the participant had tested positive for COVID-19, whether or not they had close contact with someone who had tested positive, and their vaccination status (unvaccinated, received one vaccination, received two vaccinations, or received three vaccinations).

#### 2.4.5. Beck depression inventory second edition

The Chinese version of the BDI-II consists of 21 questions based on the edition published in 1996. Each item is answered on a 4-point scale ranging from 0 to 3. The BDI-II had good factorial validity and reliability (Cronbach’s α = 0.922, 0.938, and 0.943 in P1, P2, and P3, respectively). Participants’ levels of depression were determined based on established criteria in the literature: normal (<14), mild depression (14–19), moderate depression (20–28), or major depression (29–63) (Aaron Beck, 1996).

Our online survey contained a total of 41 questions (20 demographic and health-related questions, and 21 BDI-II questions). All participants were required to complete all of these items with reference to three different time points: before quarantine, at the early stage of the quarantine, and at the late stage of the quarantine.

### 2.5. Statistical analysis

First, we calculated the median score for all items and quantitative covariates, along with interquartile ranges (IQRs). We calculated medians instead of means because the data were mostly not normally distributed. After the distribution of BDI-II scores was found to be non-normal, we log-transformed the BDI-II scores before further analyses. Unless noted otherwise, all ANOVA, *t*-tests, and regression analyses were performed on log-transformed BDI-II scores. To explore the potential predictive factors for changes in depression, a linear regression model was constructed, with changes in depressive symptoms as a dependent variable and quarantine area, relationship with parents, duration of video games/physical exercise per day, and satisfaction with the food supplied as predictive variables. To explore the effects of romantic relationships and vaccination on depression, repeated-measures ANOVAs were conducted with log-transformed BDI-II scores as the dependent variable, quarantine period (Period 1, 2, or 3) as a within-subjects variable, and relationship status (with a partner vs. single) or vaccination status as a between-subjects variable. A *post hoc* independent *t*-test was conducted to further examine group differences in depression in each stage. Coefficients of the predictive factors are presented as odds ratios (ORs) with 95% CIs. Statistical analysis was performed using SPSS version 23. The significance threshold was set at *p* = 0.05.

## 3. Results

### 3.1. A rapid increase in depressive symptoms after on-campus quarantine

We collected 274 valid sets of responses, after excluding two questionnaires with missing values. All descriptive statistics on demographics and other risk factors (e.g., daily physical exercise time) are summarized in Table 1.

**Table 1:** Factors included in the logistic regression model as predictors of BDI-II score.

Characteristic	Students, No. (%)			*p*	95% CI
**Gender**					
Male	111/274 (40.5%)			0.75	1.11 (0.59–2.11)
Female	163/274 (59.5%)			NA	NA
**Level of study**					
Undergraduate	181/274 (66.1%)			0.20	0.50 (017–1.45)
Graduate	93/274 (33.9%)			NA	NA
**COVID-19 background**				0.02*	4.10 (1.25–13.35)
COVID positive	1/274 (0.4%)			NA	NA
Close contact	22/274 (8.0%)			NA	NA
Other	251/274 (91.6%)			NA	NA
**Vaccination status**					
Unvaccinated	6/259 (2.3%)			0.83	0.83 (0.15–4.63)
Vaccinated	253/259 (97.7%)				
**Relationship status**					
With partner	106/274 (38.7%)			0.007^**^	0.42 (0.22–0.79)
Single	168/274 (61.3%)			NA	NA
**Stress relief activities**	**Period 1**	**Period 2**	**Period 3**		
Contact with parents	8.0	19.2	13.8	0.65	0.71 (0.17–3.06)
Contact with relatives	3.3	1.5	1.8	0.20	NA
Contact with friends	20.3	22.5	27.2	0.30	NA
Internet use	20.3	18.5	14.9	0.41	NA
Games	12.0	9.0	9.4	0.04^*^	NA
Music	10.9	8.3	9.0	0.73	0.75 (0.15–3.73)
Sports	20.3	5.4	8.0	0.54	NA
Other	4.7	0.4	0.4	0.65	0.76 (0.50–1.13)

***p < 0.001, **p < 0.01, *p < 0.05.

**Figure 1:**
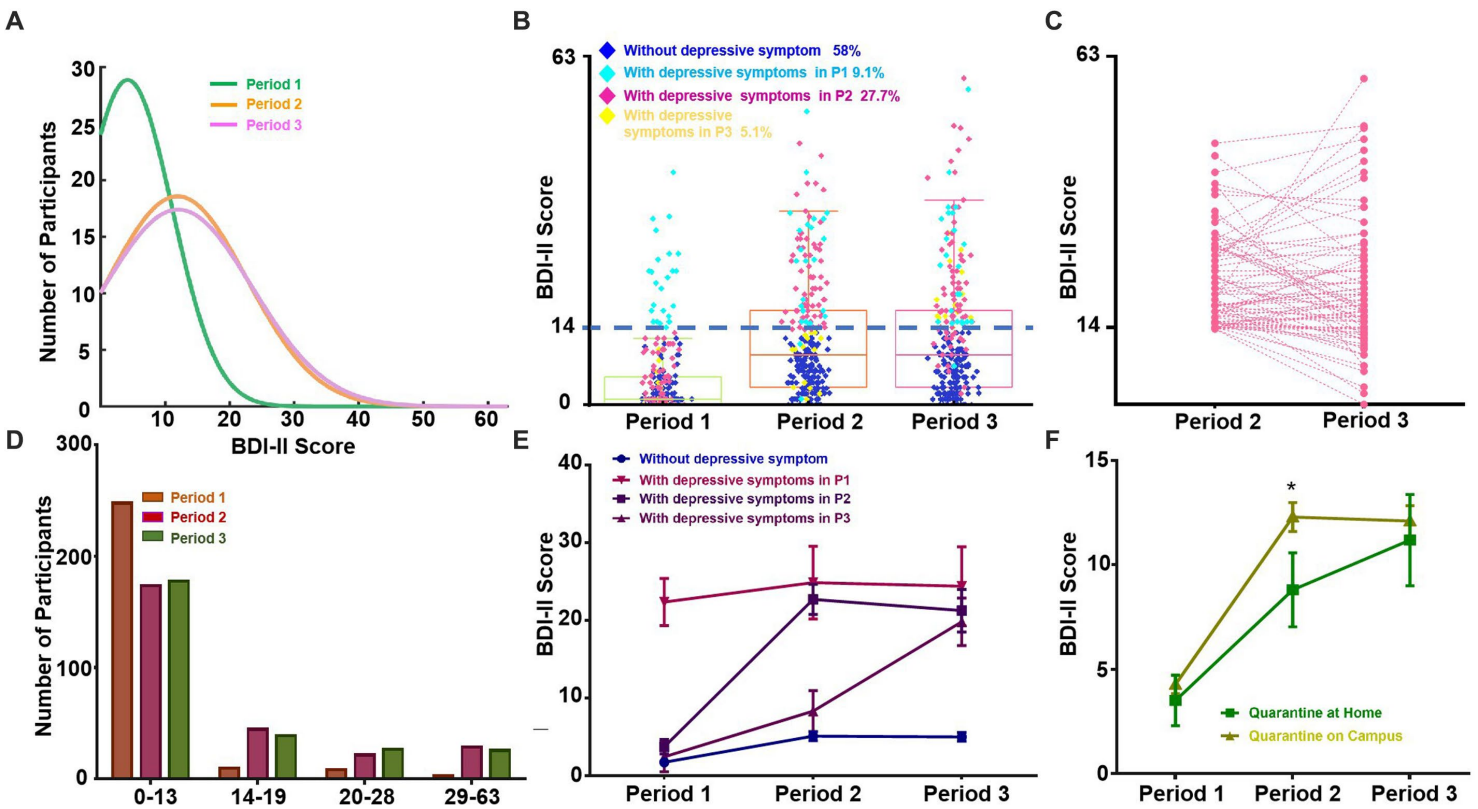
Quarantine-related changes in the distributions of depressive symptoms. **(A)** Normal distribution fit of BDI-II scores in three phases of the quarantine. The green, orange, and pink lines represent the distribution of BDI-II scores in P1, P2, and P3, respectively. **(B)** BDI-II scores in three phases of campus quarantine. Boxes represent interquartile ranges, from the lower quartile/25th percentile (bottom of the box) to the upper quartile/75th percentile (top of the box), with the median represented by the line within the box. Dark blue, light blue, pink, and yellow points represent students who never experienced depressive symptoms, those who exhibited depressive symptoms in P1, those who exhibited depressive symptoms in P2, and those who exhibited depressive symptoms in P3, respectively. **(C)** Change in BDI-II score among students without depressive symptoms in P1 and with depressive symptoms in P2. **(D)** The number of students in each of four BDI-II score subgroups in each of the three periods of time. **(E)** BDI-II scores (mean ± 95% CI) in each of the three periods among four subgroups: students without depressive symptoms (dark blue), students with depressive symptoms in P1 (pink), students with depressive symptoms in P2 (dark purple), and students with depressive symptoms in P3 (light purple). **(F)** BDI-II scores (mean ± SEM) of participants quarantined on campus vs. at home in each of the three periods. *p < 0.05, **p < 0.01, ***p < 0.001, ****p < 0.0001; otherwise not significant. P1: Period 1; P2: Period 2; P3: Period 3.

We performed a one-way repeated-measures ANOVA with log-transformed BDI-II score as the dependent variable and quarantine period (Period 1, 2, or 3) as a within-subjects variable. There was a main effect of quarantine period (*F* (2, 528) = 140.08, *p* < 0.001, *η2* = 0.35). *Post hoc* analyses revealed that depression severity was significantly increased in Period 2 (mean = 11.46, SE = 0.64) compared to Period 1 (mean = 3.95, SE = 0.40, paired *p* < 0.001). However, comparable depressive symptoms were observed in Periods 2 and 3 (mean = 11.40, SE = 0.67, *p* = 0.99), suggesting that students’ depressive symptoms did not recover immediately with broadened access to activities (Figure 1A).

We additionally plotted raw BDI-II score distributions for the three time periods. We divided the participants into two subgroups: students with depressive symptoms (BDI-II score ≥ 14) and students without depressive symptoms (BDI-II score < 14). We observed that the portion of students with depressive symptoms started at 9.1% in Period 1 and rapidly increased to 36.1% during Period 2 (Wilcoxon matched-pairs signed-rank test, *p* < 0.001), whereas no significant difference was observed between Periods 2 and 3 (34.67%) (Wilcoxon matched-pairs signed-rank test, *p* = 0.954; Figures 1A,B). Further analysis was conducted to examine participants without depressive symptoms in Period 1 who exhibited depressive symptoms in Period 2 (Figure 1C). Of these, 22.37% recovered in Period 3 (BDI-II score < 14).

According to the categorization criteria of the BDI-II, we further divided students into four subgroups according to depression symptoms: no depression (<14), mild depression (14–19), moderate depression (20–28), and major depression (>28; Figure 1D). The proportion of students in all three depression subgroups increased from Period 1 (90.88% no depression, 4.01% mild, 3.65% moderate, and 1.46% major) to Period 2 (63.87% no depression, 16.79% mild, 8.39% moderate, and 10.95% major; Wilcoxon matched-pairs signed-rank test, *p*s < 0.001), but the proportions in each subgroup only fluctuated from Period 2 to Period 3 (65.33% no depression, 14.60% mild, 10.22% moderate, and 9.85% major; Wilcoxon matched-pairs signed-rank test, *p*s ≥ 0.05). A one-way repeated-measures ANOVA was applied over the subgroups of participants who experienced depressive symptoms at each time period. A main effect of quarantine period on depressive symptoms was evident in all groups, except for those who were already experiencing depressive symptoms before the university quarantine (*N* = 25; one-way repeated-measures ANOVA, *F* (2, 48) = 1.90, *p* = 0.16, *η^2^* = 0.07) (Figure 1E, line with downward-pointing triangle symbol). As the *post hoc* analysis revealed, depressive symptoms dramatically increased in Periods 2 and 3 (Figure 1E, lines with square and upright triangle symbols) among participants who did not show depressive symptoms prior to the university quarantine (all *p*s ≤ 0.001). For example, significantly elevated depressive symptoms were observed in Period 2 (mean = 5.10, SE = 0.29) compared with Period 1 (mean = 1.73, SE = 0.21, paired *t*(157) = −11.76, *p* < 0.001, Cohen’s *d* = 0.38, Bonferroni-corrected).

Regarding changes in depressive symptoms from Period 2 to Period 3, BDI-II scores continued to increase dramatically in a relatively small proportion of students (*N* = 14; 5.1%), reaching mild depressive levels (BDI-II scores >13; Period 2: mean = 8.30, SE = 1.21; Period 3: mean = 19.80, SE = 1.41; paired *t*(13) = −4.71, *p* < 0.001, Cohen’s *d* = 0.72, Bonferroni-corrected). No significant difference in BDI-II scores between Periods 2 and 3 (both paired *t*s ≤ 1.20, *p*s ≥ 0.24, Cohen’s *d* ≤ 0.07, Bonferroni-corrected) was found for the other 258 students (94.89%). For most students, no increase in depressive symptoms was observed, and depressive symptoms decreased numerically in Period 3 (never depressed: mean = 5.10, SE = 0.29; depressed in Period 2: mean = 22.68, SE = 0.97) relative to Period 2 (never depressed: mean = 5.00, SE = 0.29; depressed in Period 2: mean = 21.24, SE = 1.37).

Across all 274 participants, we also compared students who were under quarantine on campus with those who were at home (Figure 1F). Following the university quarantine in Period 2, depressive symptoms were significantly higher (*p* < 0.05, Mann–Whitney *U*-test) among students who were quarantined on campus (*N* = 245; Period 2: mean = 12.29, SE = 0.69; Period 3: mean = 12.11, SE = 0.73) than among those at home (*N* = 29; Period 2: mean = 8.80, SE = 1.77; Period 3: mean = 11.19, SE = 2.19).

Overall, the results revealed that there was a dramatic increase in depressive symptoms in the early phase of the university quarantine; in contrast, during the subsequent middle phase of quarantine, there was no further increase in level of depression among a large proportion of students (94.89%).

### 3.2. Factors affecting depression changes during quarantine

We constructed separate linear regression models to explore potential predictors of the changes in depressive symptoms in the early phase of quarantine (Period 2 vs. Period 1) and the middle phase (Period 3 vs. Period 2). In each regression model, the log-transformed change in BDI-II score was set as the dependent variable, with quarantine area, satisfaction with the food supplied, parental bonding, and time spent on video games/physical exercise per day as predictors. The statistical summary of the regression analysis is shown in Table 2. Critically, quarantine area (which reflects the nature of quarantine) was found to be a significant predictor of changes in depression severity in Period 2 (*β* = 0.23, *p* < 0.001), but was not a predictor of subsequent changes in depression in Period 3 (*β* = 0.07, *p* = 0.214). Notably, amount of time spent on video games was a negative predictor of changes in depressive symptoms in Period 2 (*β* = −0.23, *p* < 0.001). In addition, a shorter amount of time spent on physical exercise per day and satisfaction with the food supplied were positive predictors of changes in depression severity in both Period 2 (exercise: *β* = 0.11, *p* = 0.036; food satisfaction: *β* = 0.29, *p* < 0.001) and Period 3 (exercise: *β* = 0.18, *p* = 0.003; food satisfaction: *β* = 0.28, *p* < 0.001), reflecting students’ concerns and the vital roles of food and physical exercise in coping with depression during university quarantine.

**Table 2:** Factors associated with changes in BDI-II score according to the linear regression model.

Variables included in the model		Overall fit index		Significance of the regression coefficient			
Outcome variable	Predictors	*R*	*R* ^2^	*β*	LLCI	ULCI	*p* value
Δt1 (Period 2 – Period 1)	Quarantine area	0.54	0.29	0.23	0.41	1.09	<0.001^***^
	Parental bonding			−0.03	−0.57	0.31	0.554
	Video games			−0.23	−0.95	−0.35	<0.001^***^
	Physical exercise			0.11	0.01	0.38	0.036^*^
	Satisfaction with food supplied			0.29	0.40	0.86	<0.001^***^
Δt2 (Period 3 – Period 2)	Quarantine area	0.37	0.14	0.07	1.18	4.40	0.214
	Parental bonding			0.02	−3.15	1.37	0.674
	Video games			−0.05	−0.72	1.80	0.361
	Physical exercise			0.18	0.05	1.87	0.003^**^
	Satisfaction with food supplied			0.28	0.78	1.55	<0.001^***^

***p < 0.001, **p < 0.01, *p < 0.05.

### 3.3. Higher BDI-II scores among students in a romantic relationship

**Figure 2:**
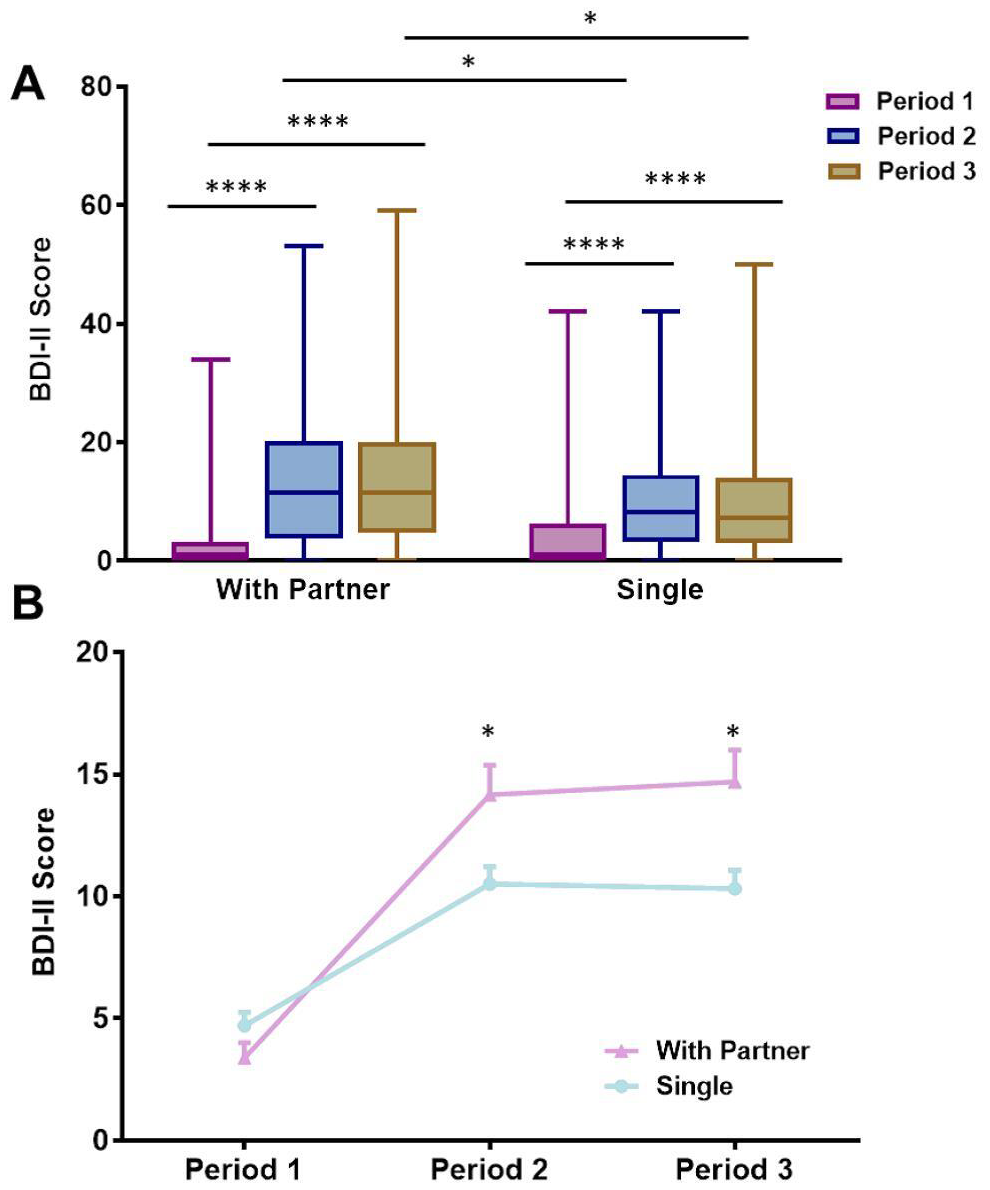
The effect of having a romantic relationship on depression during quarantine. **(A)** BDI-II scores of students with a partner vs. single students in three periods of quarantine. Boxes represent the lower quartile/25th percentile (bottom of the box), upper quartile/75th percentile (top of the box), and median (line within the box). **(B)** Median change in BDI-II scores over three periods. *p < 0.05, **p < 0.01, ***p < 0.001, ****p < 0.0001; otherwise not significant.

We conducted a repeated-measures ANOVA to further examine the influence of quarantine period and romantic relationships on depressive symptoms, with BDI-II scores as a within-subjects variable (Period 1, 2, or 3) and romantic relationship (with partner vs. without partner) as a between-subjects variable. We predicted that the influence of quarantine would be evident in both groups, but a romantic relationship would provide some protection against the influence of quarantine on depressive symptoms. As expected, a main effect of quarantine period was present (*F* (2, 526) = 164.95, *p* < 0.001, *η*^2^ = 0.39), suggesting that there was a significant change in depressive symptoms according to quarantine period among students both with and without a romantic relationship. Interestingly, although a main effect of having a romantic relationship was not evident (*F* (1, 263) = 3.66, *p* = 0.06, *η*^2^ = 0.01), a significant interaction was observed between having a romantic relationship and quarantine period (*F* (2, 536) = 17.53, *p* < 0.001, *η*^2^ = 0.06). There was no significant difference in BDI-II scores between single individuals and those with partners in Period 1 (without partner: mean = 4.70, SE = 0.55; with partner: mean = 3.39, SE = 0.63). However, the BDI-II scores of students with a partner were significantly higher than those of students without a partner in Periods 2 and 3 (Period 2: without partner: mean = 10.51, SE = 0.71; with partner: mean = 14.16, SE = 1.21. Period 3: without partner: mean = 10.32, SE = 0.75; with partner: mean = 14.69, SE = 1.30). Across all time points, depressive symptoms were numerically higher among single participants (mean = 4.5, SE = 0.53, *p* = 0.13) than among those with a partner (mean = 3.00, SE = 0.56). However, after the university quarantine, BDI-II scores were significantly higher among individuals with a romantic relationship (*N* = 106) than among those without a romantic relationship (*N* = 168); this difference was significant in Period 2 (*t*(263) = 2.50, *p* = 0.01, Cohen’s *d* = 0.38) and Period 3 (*t*(263) = 3.05, *p* = 0.003, Cohen’s *d* = 0.34), while no significant group difference was observed in the period before quarantine (*t* (263) = −1.85, *p* = 0.07, Cohen’s *d* = 0.02; Figure 2).

### 3.4. Vaccination and depression

Studies have shown that vaccination against infectious diseases may lead to a reduction in individuals’ psychological distress. We examined the effects of COVID-19 vaccination on psychological protection against depression. COVID-19 vaccination (vaccinated vs. unvaccinated) was taken into account in the preliminary analysis (see Table 1). However, as the number of participants who had not been administered the vaccine was extremely small (*N* = 6), we do not report the corresponding results in this article (see Supplementary Figure S1).

## 4. Discussion

This study aimed to investigate the acute mental health problems associated with the pandemic among college students and the factors that mitigated negative emotions during quarantine. The findings were as follows. First, a notable increase in depressive symptoms was observed in university students after the introduction of a stringent quarantine. Second, depression symptoms did not recover immediately when access to activities was broadened and the quarantine area increased. Third, the impact of a sudden change in living space on psychological resilience was more evident among students who were in a romantic relationship than among single individuals. Fourth, lower satisfaction with the food supplied and amount of time spent engaging in physical exercise per day were found to have a positive impact on changes in depressive severity.

Quarantine is always associated with negative mental health outcomes. In this study, a notable increase in level of depression was observed in university students after the introduction of a stringent quarantine. This finding is consistent with previous observations (6, 7, 11, 19–21) that students become more depressed after quarantine. Furthermore, we took into account the duration of the quarantine. Several studies have shown that a longer quarantine is associated with poorer mental health outcomes. For example, Hawryluck et al. (22) found that individuals who were quarantined for more than 10 days had more severe posttraumatic stress symptoms than those who were quarantined for less than 10 days. However, studies have also shown that even a short period of quarantine is predictive of depression symptoms. Bai et al. (23) reported that a 9-day quarantine resulted in acute stress disorder that lasted 3 years. We examined three periods that represented different quarantine statuses: Period 1 was the prequarantine period, Period 2 was during the quarantine, and Period 3 was the postquarantine period. We found that depression symptoms rapidly increased after 2 weeks of quarantine and did not recover with access to a broadened sphere of activities and a larger area in Period 3. Therefore, even a short-term quarantine would be likely to interfere with long-term functioning. After a period of quarantine, schools still need to pay attention to students’ mental health and guide them toward effective ways of relieving their psychological stress.

Furthermore, we examined the effects of stressors during quarantine while including quarantine period as a main effect. Stressors during a quarantine include the duration of the quarantine, the fear of infection with the virus for oneself and one’s loved ones, inadequate supplies, and the suspension of social public services. Previous studies have highlighted the fact that social relationships play a key role during quarantine. For example, living with family, children, roommates, or alone has been found to lead to different mental health outcomes (6, 13, 17, 19, 24). In the present study, more severe depressive symptoms were observed among participants who had a partner. The findings of recent reviews have indicated that during the COVID-19 pandemic, quarantine could undermine close relationships, enhance them, or leave them unchanged (25, 26). Relationship quality, including closeness, assistance, emotional security, and support, plays a crucial role in close relationships, which may be interrupted by quarantine. The campus quarantine interrupted these relationships, contributing to students’ psychological distress. The frequency of joint activities decreased greatly during the quarantine period, and being deprived of contact with a loved one could lead to relationship dissatisfaction and might cause more severe depressive symptoms. In contrast, single individuals had more time to themselves before the quarantine. Despite the increase in their solitary time after the introduction of the quarantine, the change in the pace of their lives was not as great compared to that of people with partners. This effect was also in evidence when we compared the group quarantined in school to the group at home.

A linear regression analysis was conducted in the present study and revealed four predictors of changes in depressive symptoms during quarantine: the quarantine area, amount of time spent playing video games, amount of time spent engaging in physical exercise, and satisfaction with food. We found that smaller quarantine area, less time spent on physical exercise, and less satisfaction with the food supplied during quarantine led to increased depression levels. In contrast, more time spent on video games per day was a negative predictor of increased depressive symptoms. Spending longer playing video games may prevent the development of negative emotions to some extent (27). This could be because video games were able to distract students’ attention from COVID-19 and its associated issues, which may have relieved their depression (28). Meanwhile, students were able to communicate with others while playing video games, which is a good way to reduce stress. Given the widespread popularity of video games, universities could guide students toward appropriate use of video games to better support them during the current COVID-19 pandemic. Additionally, many studies have shown that exercise can bring about physiological changes that can improve mood state and reduce stress and anxiety levels (24, 29, 30). Finally, many individuals increase their intake of highly palatable foods during times of stress, which can reduce physiological and psychological measures of stress; in contrast, a limited and less satisfying food selection during the quarantine could cause them to be unable to relieve stress through food.

To understand the benefits of vaccination for mental health, we compared the BDI-II scores of people who had received the vaccine with those who had not. However, the number of the population who had not been administered any vaccinations was extremely small (*N* = 6). Several studies have indicated that vaccination is correlated with a decline in poor mental health. (31) found that vaccination provided additional psychological benefits, in addition to decreasing the risk of severe symptoms, by reducing perceived risk and fear. Jaiswal et al. (32) showed that psychological immunity, such as environmental mastery, is strongly correlated with mental and physical health. Further studies may be needed that consider vaccination as a psycho-protective factor.

## 5. Limitations

First, the number of participants was relatively limited. The data in the present study represent approximately 1% of all students at SJTU. Therefore, caution is warranted in applying the present results to the entire population. Second, in the present study, the survey was administered to participants on a single occasion, on which they were required to rate their depressive symptoms for three different periods. It would be more accurate to ask participants about their current emotional status at three different times. Third, we assessed only changes in depressive symptoms in this study; other mental health issues should also be investigated, such as anxiety symptoms and suicidal thoughts. In addition, a longitudinal or cohort study could be performed to determine how mental health status changes over time until the release of campus-wide quarantine, and to identify the factors associated with mental health status. Furthermore, posttraumatic growth for young adults (33) and employment (34) for students approaching graduation are crucial. More specific studies should be designed for those groups.

## 6. Conclusion

In conclusion, the present study revealed a notable increase in depression among university students after the introduction of a 2-week quarantine. No perceptible reversal of depressive symptoms over time was observed, even when students were allowed to re-enter the campus for essential activities. With regard to students who are in a relationship, providing ways to take physical exercise and relax and improving food supplies should be considered as strategies when young people are quarantined.

## Data availability statement

The original contributions presented in the study are included in the article/Supplementary material, further inquiries can be directed to the corresponding authors.

## Ethics statement

The studies involving human participants were reviewed and approved by Shanghai Jiao Tong University. The patients/participants provided their written informed consent to participate in this study.

## Author contributions

WL conceived and supervised the project. YK, DM, ZL, BZ, SZ, YL, MS, R-YZ, and BZ conceived and designed the survey questions. YK, DM, ZL, and BZ analyzed the data and drafted the manuscript. All authors contributed to the article and approved the submitted version.

## Funding

This study was supported by the Shanghai Education Commission Research and Innovation Program (2019-01-07-00-02-E00037), the National Key Research and Development Program of China (2018YFE0126700), the Program of Shanghai Subject Chief Scientist (17XD1401700), the Ministry Key Project (GW0890006), Shanghai Municipal Commission of Science and Technology Program (21dz2210100), the “111” Program of Higher Education Discipline Innovation, Shanghai Jiao Tong University Scientific and Technological Innovation Funds (to WL), and the Research Foundation of Shanghai Jiao Tong University (YG2022QN106) (to BZ).

## Conflict of interest

The authors declare that the research was conducted in the absence of any commercial or financial relationships that could be construed as a potential conflict of interest.

## Publisher’s note

All claims expressed in this article are solely those of the authors and do not necessarily represent those of their affiliated organizations, or those of the publisher, the editors and the reviewers. Any product that may be evaluated in this article, or claim that may be made by its manufacturer, is not guaranteed or endorsed by the publisher.

## Footnotes

^1^ www.wjx.cn

## References

[1] World Health Organization. WHO Director-General's Opening Remarks at the Media Briefing on COVID-19 - 11 March 2020; (2020). Available at: https://www.who.int/dg/speeches/detail/whodirector-general-s-opening-remarks-at-the-media-briefing-oncovid-19---25-may-2020. (Accessed 24 June 2020).

[2] Brooks SK Webster RK Smith LE Woodland L Wessely S Greenberg N. The psychological impact of quarantine and how to reduce it: rapid review of the evidence. Lancet. (2020) 395:912–20. doi: 10.1016/S0140-6736(20)30460-8, PMID: 32112714 PMC7158942

[3] Quadros S Garg S Ranjan R Vijayasarathi G Mamun MA. Fear of COVID 19 infection across different cohorts: a scoping review. Front Psych. (2021) 12:708430. doi: 10.3389/fpsyt.2021.708430, PMID: 34557117 PMC8453018

[4] Dutheil F Mondillon L Navel V. PTSD as the second tsunami of the SARS-Cov-2 pandemic. Psychol Med. (2021) 51:1773–4. doi: 10.1017/S0033291720001336, PMID: 32326997 PMC7198460

[5] Burki T. Dynamic zero COVID policy in the fight against COVID. Lancet Respir Med. (2022) 10:e58–9. doi: 10.1016/S2213-2600(22)00142-4, PMID: 35460629 PMC9020804

[6] Fancourt D Steptoe A Bu F. Trajectories of anxiety and depressive symptoms during enforced isolation due to COVID-19 in England: a longitudinal observational study. Lancet Psychiatry. (2021) 8:141–9. doi: 10.1016/S2215-0366(20)30482-X, PMID: 33308420 PMC7820109

[7] Tran HTT Nguyen MH Pham TTM Kim GB Nguyen HT Nguyen NM. Predictors of eHealth literacy and its associations with preventive behaviors, fear of COVID-19, anxiety, and depression among undergraduate nursing students: a cross-sectional survey. Int J Environ Res Public Health. (2022) 19:3766. doi: 10.3390/ijerph19073766, PMID: 35409448 PMC8997661

[8] Marroquin B Vine V Morgan R. Mental health during the COVID-19 pandemic: effects of stay-at-home policies, social distancing behavior, and social resources. Psychiatry Res. (2020) 293:113419. doi: 10.1016/j.psychres.2020.113419, PMID: 32861098 PMC7439968

[9] Racine N Mcarthur BA Cooke JE Eirich R Zhu J Madigan S. Global prevalence of depressive and anxiety symptoms in children and adolescents during COVID-19: a meta-analysis. JAMA Pediatr. (2021) 175:1142–50. doi: 10.1001/jamapediatrics.2021.2482, PMID: 34369987 PMC8353576

[10] Ochnik D Rogowska AM Kusnierz C Jakubiak M Schutz A Held MJ. Mental health prevalence and predictors among university students in nine countries during the COVID-19 pandemic: a cross-national study. Sci Rep. (2021) 11:18644. doi: 10.1038/s41598-021-97697-3, PMID: 34545120 PMC8452732

[11] Son C Hegde S Smith A Wang X Sasangohar F. Effects of COVID-19 on college Students' mental health in the United States: interview survey study. J Med Internet Res. (2020) 22:e21279. doi: 10.2196/21279, PMID: 32805704 PMC7473764

[12] Tang W Hu T Hu B Jin C Wang G Xie C. Prevalence and correlates of PTSD and depressive symptoms one month after the outbreak of the COVID-19 epidemic in a sample of home-quarantined Chinese university students. J Affect Disord. (2020) 274:1–7. doi: 10.1016/j.jad.2020.05.009, PMID: 32405111 PMC7217769

[13] Wathelet M Duhem S Vaiva G Baubet T Habran E Veerapa E. Factors associated with mental health disorders among university students in France confined during the COVID-19 pandemic. JAMA Netw Open. (2020) 3:e2025591. doi: 10.1001/jamanetworkopen.2020.25591, PMID: 33095252 PMC7584927

[14] Daly M Robinson E. Psychological distress and adaptation to the COVID-19 crisis in the United States. J Psychiatr Res. (2021) 136:603–9. doi: 10.1016/j.jpsychires.2020.10.035, PMID: 33138985 PMC7588823

[15] Wu P Katic BJ Liu X Fan B Fuller CJ. Mental health service use among suicidal adolescents: findings from a U.S. national community survey. Psychiatr Serv. (2010) 61:17–24. doi: 10.1176/ps.2010.61.1.17, PMID: 20044413

[16] Chen F Zheng D Liu J Gong Y Guan Z Lou D. Depression and anxiety among adolescents during COVID-19: a cross-sectional study. Brain Behav Immun. (2020) 88:36–8. doi: 10.1016/j.bbi.2020.05.061, PMID: 32464156 PMC7247496

[17] Li S Wang Y Xue J Zhao N Zhu T. The impact of COVID-19 epidemic declaration on psychological consequences: a study on active Weibo users. Int J Environ Res Public Health. (2020) 17:2032. doi: 10.3390/ijerph17062032 32204411 PMC7143846

[18] Li Y Zhao J Ma Z Mcreynolds LS Lin D Chen Z. Mental health among college students during the COVID-19 pandemic in China: a 2-wave longitudinal survey. J Affect Disord. (2021) 281:597–604. doi: 10.1016/j.jad.2020.11.109, PMID: 33257043

[19] Cao W Fang Z Hou G Han M Xu X Dong J. The psychological impact of the COVID-19 epidemic on college students in China. Psychiatry Res. (2020) 287:112934. doi: 10.1016/j.psychres.2020.112934, PMID: 32229390 PMC7102633

[20] Husky MM Kovess-Masfety V Gobin-Bourdet C Swendsen J. Prior depression predicts greater stress during Covid-19 mandatory lockdown among college students in France. Compr Psychiatry. (2021) 107:152234. doi: 10.1016/j.comppsych.2021.152234, PMID: 33706216

[21] Shi L Lu ZA Que JY Huang XL Lu QD Liu L. Long-term impact of COVID-19 on mental health among the general public: a Nationwide longitudinal study in China. Int J Environ Res Public Health. (2021) 18:8790. doi: 10.3390/ijerph18168790, PMID: 34444539 PMC8393580

[22] Hawryluck L Gold WL Robinson S Pogorski S Galea S Styra R. SARS control and psychological effects of quarantine, Toronto, Canada. Emerg Infect Dis. (2004) 10:1206–12. doi: 10.3201/eid1007.030703, PMID: 15324539 PMC3323345

[23] Bai Y Lin CC Lin CY Chen JY Chue CM Chou P. Survey of stress reactions among health care workers involved with the SARS outbreak. Psychiatr Serv. (2004) 55:1055–7. doi: 10.1176/appi.ps.55.9.1055, PMID: 15345768

[24] Broman-Fulks JJ Berman ME Rabian BA Webster MJ. Effects of aerobic exercise on anxiety sensitivity. Behav Res Ther. (2004) 42:125–36. doi: 10.1016/S0005-7967(03)00103-7 14975776

[25] Schmid L Worn J Hank K Sawatzki B Walper S. Changes in employment and relationship satisfaction in times of the COVID-19 pandemic: evidence from the German family panel. Eur Soc. (2021) 23:S743–58. doi: 10.1080/14616696.2020.1836385

[26] Williamson HC. Early effects of the COVID-19 pandemic on relationship satisfaction and attributions. Psychol Sci. (2020) 31:1479–87. doi: 10.1177/0956797620972688, PMID: 33151125 PMC7797601

[27] Granic I Lobel A Engels RC. The benefits of playing video games. Am Psychol. (2014) 69:66–78. doi: 10.1037/a0034857 24295515

[28] Kowal M Conroy E Ramsbottom N Smithies T Toth A Campbell M. Gaming your mental health: a narrative review on mitigating symptoms of depression and anxiety using commercial video games. JMIR Serious Games. (2021) 9:e26575. doi: 10.2196/26575, PMID: 34132648 PMC8277305

[29] Abd El-Kader SM Al-Jiffri OH. Exercise alleviates depression related systemic inflammation in chronic obstructive pulmonary disease patients. Afr Health Sci. (2016) 16:1078–88. doi: 10.4314/ahs.v16i4.25 28479901 PMC5398455

[30] Bartholomew JB Morrison D Ciccolo JT. Effects of acute exercise on mood and well-being in patients with major depressive disorder. Med Sci Sports Exerc. (2005) 37:2032–7. doi: 10.1249/01.mss.0000178101.78322.dd 16331126

[31] Koltai J Raifman J Bor J Mckee M Stuckler D. COVID-19 vaccination and mental health: a difference-in-difference analysis of the understanding America study. Am J Prev Med. (2022) 62:679–87. doi: 10.1016/j.amepre.2021.11.006, PMID: 35012830 PMC8674498

[32] Jaiswal A Singh T Arya YK. "Psychological antibodies" to safeguard frontline healthcare warriors mental health against COVID-19 pandemic-related psychopathology. Front Psych. (2020) 11:590160. doi: 10.3389/fpsyt.2020.590160, PMID: 33391053 PMC7775359

[33] Cohen-Louck K. Differences in post-traumatic growth: individual quarantine, COVID-19 duration and gender. Front Psychol. (2022) 13:920386. doi: 10.3389/fpsyg.2022.920386, PMID: 35928418 PMC9344049

[34] Levy I. Stress, anxiety, and depression in times of COVID-19: gender, individual quarantine, pandemic duration and employment. Front Public Health. (2022) 10:999795. doi: 10.3389/fpubh.2022.999795, PMID: 36408032 PMC9670105

