# Supplementary figures and images for: The rapid change in mental health among college students after introduction of on-campus quarantine during the 2022 Shanghai COVID-19 lockdown
Source: Front Public Health. 2023 May 5;11:1132575. doi: 10.3389/fpubh.2023.1132575 (PMC10196357; doi:10.3389/fpubh.2023.1132575)

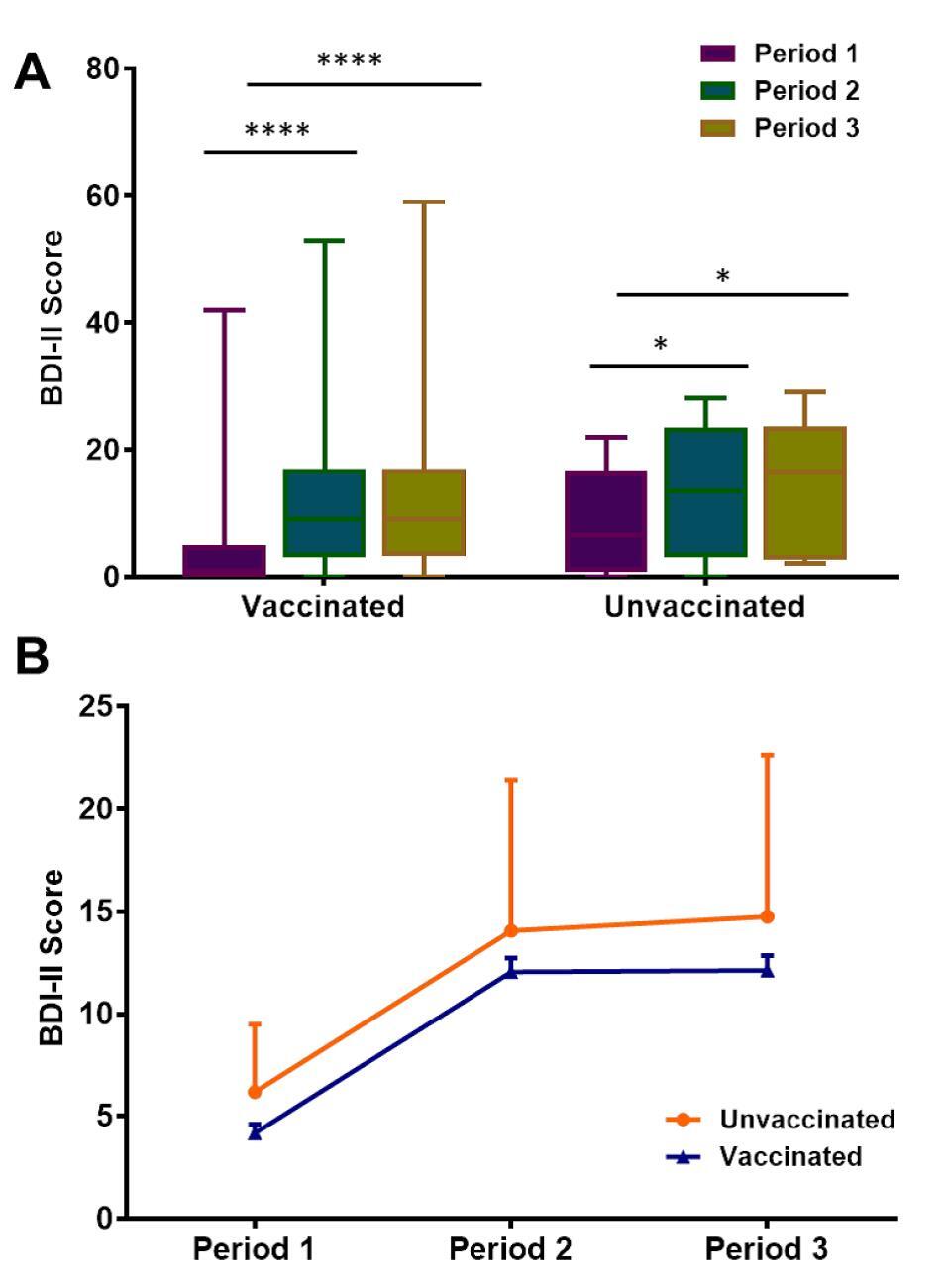

Supplement: Supplementary file 1 [file Image_1.JPEG]
